# The Role of Long Polar Fimbriae in *Escherichia coli* O104:H4 Adhesion and Colonization

**DOI:** 10.1371/journal.pone.0141845

**Published:** 2015-10-30

**Authors:** Brittany N. Ross, Maricarmen Rojas-Lopez, Roberto J. Cieza, Brian D. McWilliams, Alfredo G. Torres

**Affiliations:** 1 Department of Microbiology and Immunology, University of Texas Medical Branch, Galveston, Texas, 77555–1070, United States of America; 2 Department of Pathology and Sealy Center for Vaccine Development, University of Texas Medical Branch, Galveston, Texas, 77555–1070, United States of America; USDA-ARS-ERRC, UNITED STATES

## Abstract

A renewed interest in Shiga toxin-producing *Escherichia coli* (STEC) strains was sparked due to the appearance of an outbreak in 2011, causing 3,816 diarrheal cases and some deaths in Europe. The causative strain was classified as enteroaggregative *E*. *coli* of serotype O104:H4 that had acquired Shiga toxin genes. The ability of STEC O104:H4 to cause disease relies greatly on the bacteria’s capacity to colonize, persist, and produce Shiga toxin. However, not much is known about the colonization factors of this strain. Because long polar fimbriae (*lpf*) *lpf1* and *lpf2* operons encode important colonization factors in other STEC isolates and *E*. *coli* O104:H4 possesses both loci, we hypothesized that Lpf is required for adhesion and colonization. In this study, isogenic *lpfA1* and *lpfA2* major fimbrial subunit mutants were constructed. To determine their role in O104:H4’s virulence, we assessed their ability to adhere to non-polarized and polarized intestinal epithelial cells. The Δ*lpfA1* showed decreased adherence in both cell systems, while the Δ*lpfA2* only showed a decrease in adherence to polarized Caco-2 cells. We also tested the O104:H4 mutants’ ability to form biofilm and found that the Δ*lpfA1* was unable to form a stable biofilm. In an *in vivo* murine model of intestinal colonization, the Δ*lpfA1* had a reduced ability to colonize the cecum and large intestine, consistent with the *in vitro* data. Further, we tested the *lpfA1* mutants’ ability to compete against the wild type. We found that in the *in vitro* and *in vivo* models, the presence of the wild type O104:H4 facilitates increased adherence of the Δ*lpfA1* to levels exceeding that of the wild type. Overall, our data demonstrated that Lpf1 is one of the factors responsible for O104:H4 intestinal adhesion and colonization.

## Introduction

The 2011 European *Escherichia coli* outbreak was one of the largest associated with Shiga toxin-producing *E*. *coli* (STEC) strains ever recorded [1,2,3,4]. The epidemic started in northern Germany and extended into Europe, affecting predominantly adults [1]. This emergent strain of STEC was originally thought to be a hybrid of enteroaggregative *E*. *coli* (EAEC) and STEC, but genomic analysis revealed that the strain is an uncommon EAEC of serotype O104:H4 that had acquired phage-borne genes encoding Shiga toxin 2 (Stx2) [2,3,4,5]. Characteristic of other typical EAEC strains, O104:H4 also possesses the pAA virulence plasmid that encodes the Aggregative Adherence Fimbriae (AAF) [3,6]. In addition to this recent outbreak, the pathovar EAEC is commonly the cause of persistent diarrhea in children in developing countries and the second leading cause of traveler’s diarrhea [7,8]. With the appearance of STEC O104:H4 in other countries prior to 2011, EAEC/STEC are considered emerging pathogens by the CDC [7].

The mechanism of pathogenesis for this newly described STEC O104:H4 strain is still fairly unknown, using information about EAEC, it is predicted that the bacteria will bind to the intestinal surface and induce increased intestinal mucus secretion and goblet cell pitting [9]. The excess mucus traps bacteria and aids in the formation of the aggregative phenotype (biofilm), which is believed to enhance virulence and persistence [10,11]. Once establishment of the bacteria in the intestine has occurred, secretion of enterotoxins and cytotoxins follows [7]. The infection ultimately results in destructive lesions, shortening of the villi, hemorrhagic necrosis of the villous tips, and a mild inflammatory response with edema and mononuclear infiltration of the submucosa [9,12]. Because this bacterium is able to produce a biofilm *in vivo*, the best way to prevent and control the infection is to target colonization, as well as potential re-colonization that might occur when bacteria disperses from a mature biofilm [11,13,14].

Previous studies on EAEC strains showed that colonization and the typical brick stacking phenotype was a result of aggregative adherence fimbriae I (AAF/I) found on the pAA plasmid [15,16,17]. Recently, there have been conflicting findings questioning whether AAF/I fimbriae is responsible for colonization. One paper showed that pAA, which contains the aggregative adherence (*agg*) operon, does not affect colonization of infant rabbits [6]. Further, it has also been shown that the pAA plasmid is lost during infection suggesting that its importance is transient [18]; however, it has been suggested that the presence of the fimbriae can promote the translocation of Stx2a across the epithelia [19]. Looking to other potential players in adherence of STEC strains, O104:H4 lacks intimin, which is the key adhesin in the Locus of Enterocyte Effacement (LEE)-positive STEC strains, such as O157:H7 [5,20,21]. However, LEE-negative STEC strains are still able to adhere to tissue cultured cells, indicating that other proteins participate in the initial adhesion and further colonization of the intestinal tract [22].

Genome sequence analysis of *E*. *coli* O104:H4 showed that this strain possesses the long polar fimbriae (*lpf*) operons, previously characterized in STEC O157:H7 [21,22]. The *lpf1* operon was first identified in *Salmonella enterica* serovar Typhimurium, while *lpf2* was later identified in *E*. *coli* O157:H7 [23,24]. The *lpf1* loci consist of 5 genes (*lpfA*, *B*, *C*, *D* and *E*), while *lpf2* has 4–5 genes (*lpf A*, *B*, *C*, *D* and some strains carry a duplicated *D’*) [22]. The predicted functions of each gene-encoded protein are as follows; LpfAs proteins serve as the major fimbrial subunits, LpfBs are chaperones, LpfCs are outer membrane usher proteins, LpfDs are minor fimbrial subunits, and lastly LpfE has been described as a regulator or an additional fimbrial subunit [21].

We have previously reported that STEC Lpfs play an important role in colonization, persistence, and pathogenesis. For example, sheep and conventional pigs inoculated with STEC O157:H7 lacking both *lpf1* and *lpf2* resulted in reduced bacteria recovery in feces [25]. In neonatal gnotobiotic piglets, the STEC *lpf* double mutant resulted in fewer histopathological intestinal lesions. In 6-week cross-breed lambs and infant rabbits, *lpf1* and *lpf2* mutants each showed pronounced decrease in colonization and lower levels of bacteria recovered from feces compared to wild type [24,26,27,28]. Furthermore, O157:H7 Lpf1 and Lpf2 are associated with human intestinal tissue tropism [29] and neutrophil transmigration and inflammation [30,31].

Because O104:H4 requires initial attachment to the intestine, it is plausible to suggest that this strain depends on the Lpf fimbriae for effective attachment and colonization. Here, we constructed isogenic mutants of both *lpfA1* and *lpfA2* fimbrial major subunit genes to determine their involvement in O104:H4 colonization as well as in biofilm formation.

## Materials and Methods

### Ethics statement

All manipulations of *E*. *coli* strains were conducted in approved and certified biosafety level 2 facilities at the University of Texas Medical Branch (UTMB), and experiments were performed in accordance with standard operating practices. The animal studies were carried out in strict accordance with the recommendations in the Guide for the Care and Use of Laboratory Animals of the National Institutes of Health. The protocol (IACUC #0709042B) was approved by the Animal Care and Use Committee of the UTMB.

### Lpf conservation and distribution analysis

For analysis of the presence of both *lpf1* and *lpf2* operons, the O104:H4 2011c-3493 operon’s genes and protein sequences were aligned against EAEC 55989, O104:H4 2009EL-2050, O104:H4 C227-11, and EHEC O157:H7 EDL933 (Public reported genome sequences obtained from NCBI). Alignments where done with MUSLE and MAFFT in Geneious software version 8 (http://www.geneious.com/features/genome-alignment). The parameters for MUSCLE were as follows: max iterations-8, cluster method-UPGMB, sequencing weighting scheme-CLUSTALW. For MAFFT the parameters used were: algorithm-auto, scoring matrix-BLOSSUM30, gap penalty-1, and offset value- 0.123.

The distribution analysis was done using *lpfA* genes as query entrance against a total of 1167 public genomes obtained from NCBI that were downloaded and a megablast (fast and high similarity) was performed. The applied parameters allowed us to identify whether the down/upstream sequence contained the whole *lpf’s* operons and the query coverage taken into account was >90% and >80% of identity, respectively.

### Bacterial strain and mutant construction

All mutant strains used in this study where derived from *E*. *coli* O104:H4 strain C3493, isolated from a stool sample of a patient with HUS during the 2011 German outbreak. The bacterial sample was obtained from the Enteric Diseases Laboratory Branch, Center of Disease Control and Prevention (CDC, Atlanta, GA) and the use of these strains was approved by the UTMB Institutional BioSafety Committee (NOU 2013050). The O104:H4 strain 2050 was recovered from an outbreak in the Republic of Georgia in 2009, and was also obtained from CDC. Table 1 presents a comprehensive list of strains and primers used. We mutated the *lpfAs* genes, which encode the major fimbrial subunits and are the first genes in both *lpf* operons [24,32]. Briefly, the *lpfA1* mutant strain was constructed via PCR production of the *lpfA1* gene from O104:H4 strain C3493 using Phusion polymerase and inserted into pGEMT via SphI restriction sites. The plasmid was cut with EcoRV leading to a single cut in the *lpfA1* gene where a chloramphenicol resistance (Cm^r^) cassette was inserted. The *lpfA1*::Cm fragment was ligated into pCVD442 and transformed into *E*. *coli* SM10 (λ *pir)*. The suicide vector was introduced into the recipient strains by conjugation, and recombinants were selected by plating strains on the appropriate antibiotics and sucrose, as previously described [33]. The *lpfA2* mutant strain was constructed by the same method but the interrupted gene fragment was created using sewing PCR inserting a gentamicin (Gm^r^) resistance cassette. The fragment was inserted into pCVD442 via the XbaI restriction site. The *lpfA1 lpfA2* double mutant strain was constructed by conjugating the *lpfA1* mutant with SM10 (λ *pir)* containing pCVD44 *lpfA2*::Gm and selected for both chloramphenicol and gentamicin. The complemented *lpfA1* was generated by inserting a PCR product of wild-type *lpfA1* flanked with 1000 base pairs into pCVD442 and conjugated into the *lpfA1* mutant, follow by selecting chloramphenicol sensitive, streptomycin resistant colonies. All the mutations were confirmed by antibiotic resistance, PCR (S1 Fig), and sequencing. All the strains were evaluated for the presence and maintenance of the pAA plasmid and the presence of the *aggA* gene (encodes the major fimbrial subunit of AAF/I fimbriae) after mutagenesis. Our PCR results indicated that all our wild-type and mutant stains carry the *aggA* gene (S1C Fig).

**Table 1:** Bacterial strains, plasmids and primers used in this study.

Strains		
O104:H4 2011 c3493	Wild type strain. Isolated from a stool sample form a patient with HUS during the 2011 outbreak	Obtained from CDC
BRL1-17B	O104:H4 2011 c3494 *lpfA1*::Cm	This study
JHU-1	O104:H4 2011 c3494 *lpfA2*::Gm	This study
BRL12-17B	O104:H4 2011 c3494 *lpfA1*::Cm *lpfA2*::Gm created by conjugating SM10 (pCVD442 *lpfA1*::Cm) with JHU-1	This study
BRL1- COM28	BRL1-17B complemented by reinsertion via recombination of *lpfA1* in its native site	This study
O104:H4 2009 2050	Recovered from an outbreak in the Republic of Georgia in 2009	Obtained from CDC
Plasmids		
pCVD442 *lpfA1*::Cm	pCVD442 with *lpfA1* interrupted with a chloramphenicol cassette	This study
pCVD442 *lpfA2*::Gm	pCVD442 *lpfA2* interrupted with a gentamicin cassette	This study
pCVD442 *lpfA1* reinsertion	pCVD442 with *lpfA1* flanked with 1000 bp	This study
Primers		
lpfA1::Cm F	CTGAATGCAGTGACGTTCTTTGCAG	
lpfA1::Cm R	GTCAGCATGCCGCCAGCAACACCGA	
CmEcoRV F	GGCGATATCACCCGACGCACTTTGC	
CmEcoRV R	GCAGATATCAGGCGGGCAAGAATGTGA	
lpfA2-5' F	AAAGGTCTAGATGCCTCCATTGAACATGACATTGTG	
lpfA2-5' Gm R	ATGTCAATTCGAGTCGGGCAATTTTCAAG	
lpfA2-5' Gm F	CTTGAAAATTGCCCGAGCTCGAATTGACAT	
lpfA2-3' Gm R	CGTTGAGTGTACGTTGGAGCTCGAATTGGC	
lpfA2-3' Gm F	GCCAATTCGAGCTCCAACGTACACTCAAGC	
lpfA2-3' R	GCTTTGGAGCTCGCGTGATGGCGGAATATT	
*lpfA1* Re-insertion F	ATATCTAGATGGTGATGATTGTTTTCGGCG	
*lpfA1* Re-insertion R	ATATCTAGAGGTATTTACCGGGGATTTGCT	

### Growth curves

All bacteria were grown in LB broth overnight at 37°C, shaking at 200 rpm, and with the appropriate antibiotics. The following day, strains were diluted to an OD_600_ of 0.1, and bacteria were returned to the shaker at 37°C. Growth was monitored by OD_600_ and CFU enumeration every 30 min for 5 h (S1 Fig).

### Immunogold labeling electron microscopy

Bacterial samples were grown in LB broth overnight at 37°C, shaking at 200 rpm with the appropriate antibiotics. The following day, cultures were diluted 1:100 into DMEM or LB and grown statically without antibiotics overnight at 37°C. The next day, 1 ml of culture was centrifuged and re-suspended in 100 μl of PBS. The bacteria were allowed to adhere to Formvar-carbon-coated copper grids (200 mesh; Electron Microscopy Sciences) as previously described [24]. For immunogold labeling of Lpf1 fimbriae, bacteria were reacted with anti-LpfA *Salmonella* serum [23] as primary and 15-nm gold-labeled anti-rabbit immunoglobulin G (AuroProbe GAR G15, RPN422; Amersham Biosciences) as secondary antibody and negatively stained with 2% potassium-phosphotungstic acid (pH 6.8). Specimens were examined in a Phillips 201 electron microscope (S1E and S1F Fig).

### Non-polarized adhesion assays

Caco-2 (ATCC® HTB-37^TM^) cells were maintained at 37°C and 5% CO_2_ in complete HTB-37 medium which consists of Eagle’s Minimum Essential Medium (EMEM, GIBCO) supplemented with 2 mM glutamine, 1 mM sodium pyruvate, 1x non-essential amino acids, penicillin-streptomycin (100 U/ml, 100 μg/ml), and 10% fetal bovine serum. For adhesion assays, 12-well plates were seeded with 10^5^ cells per well and incubated as described above to achieve 80% confluence. Approximately 1 h prior to inoculation, the monolayer was washed twice with 1 ml PBS and then 1 ml medium with no supplements was added to each well. Upon inoculation, medium was removed and replaced with 1 ml medium containing 10^7^ bacterial cells (multiplicity of infection [MOI], 100) from cultures grown in EMEM. Inoculated monolayers were incubated for 3 h at 37°C and 5% CO_2_. After incubation, cells were washed three times with PBS, and then 200 μl of 0.1% Triton X-100 in PBS was added. Wells were incubated at 37°C and 5% CO_2_ until monolayers detached from the plate. Monolayers were homogenized by pipetting, and tenfold-dilutions were plated onto LB agar using the drop plate technique, as well as spread plates. The percentage of bacteria recovered was calculated as the number of CFU/ml recovered divided by the initial number of CFU/ml to account for slight variances in input between groups. The experiments were repeated 3 times in triplicate. Mean percentages of each mutant recovered were compared to the mean percentage of the wild type recovered using one-way analysis of variance followed by Tukey’s post-test when comparing more than two groups.

### Polarized epithelial cell adhesion

Caco-2 cells were seeded at a concentration of 5 x 10^5^ on the upper side of polystyrene Transwell inserts coated with collagen (3 μm pore, 12 mm filters, CORNING) in 500 μl of complete growth medium and cultured for 14 days. Caco-2 complete growth media contains EMEM (GIBCO) supplemented with 2 mM glutamine, 1 mM sodium pyruvate, 1x non-essential amino acids, penicillin-streptomycin (100 U/ml, 100 μg/ml), and 10% fetal bovine serum. The basolateral side of the insert is filled with 1.2 ml of complete growth medium. Every other day, the medium was replaced with fresh complete medium up to 14 days. The integrity of the cell monolayer was measured by transepithelial resistance (TER) before and after the experiments with an STX2 electrode/EVOM^2^ epithelial voltammeter (World Precision Instruments). Approximately 1 h prior to inoculation, the transwell filters were washed twice with 1 ml of PBS and 1 ml of medium with no supplements was added to each well. Upon inoculation, medium on the upper side of the transwell was removed and replaced with 500 μl media containing 5 x 10^7^ bacterial cells (MOI 100). Inoculated wells were incubated for 3 h at 37°C and 5% CO_2_. At 1, 2, and 3 h, 20 μl of the basolateral medium was removed, serially diluted, and plated to monitor translocation of bacteria. After incubation, cells were washed 3x with PBS, and then 200 μl of 0.1% Triton X-100 in PBS was added to each well. Plates were incubated at 37°C and 5% CO_2_ for 15 min. The monolayers were homogenized by pipetting, and ten-fold dilutions were plated onto LB agar using the drop plate technique, as well as spread plates. The percentage of bacteria recovered was calculated as the number of CFU/ml recovered divided by the initial number of CFU/ml to account for slight variances in input between groups. The experiments were repeated 2 times in triplicate. Mean percentages of each mutant recovered were compared to the mean percentage of the wild type recovered using one-way analysis of variance followed by Tukey’s post-test when comparing more than two groups.

### Biofilm assay

The protocol used was adapted from O’Toole [34]. Briefly, isolates were grown overnight in LB and the following day cultures were diluted to an OD_600_ = 1.0 with LB followed by a 1:100 dilution into Dulbecco’s Modified Eagle’s Medium with 0.45% glucose (DMEM, GIBCO). Non-tissue culture polyvinyl chloride plates (BD, 353911) were inoculated with 125 μl and incubated aerobically at 37°C and 5% CO_2_ for 24 h. The plates were then washed 3x with PBS and stained with 0.1% crystal violet (Sigma, C3886) for 10 min. Wells were washed twice and dried for 24 h. To solubilize the bound crystal violet, 125 μl of 30% acetic acid was added to each well and incubated for 10 min. The biofilm biomass was estimated at OD_550._ Alternatively, the wells incubated for 24 h were washed 3x, and the biofilm was re-suspended by vigorous pipetting. The bacterial suspension were then serially diluted and plated to determine the number of bacteria confined in the biofilm. All data was analyzed by using one-way analysis of variance followed by Tukey’s post-test analysis when comparing more than two groups.

For further biofilm analysis, isolates were grown overnight in LB and the following day diluted to an OD_600_ = 1.0 with LB with subsequent 1:100 dilution into DMEM with 0.45% glucose (GIBCO). One ml of culture was plated in a 12-well plate containing a glass cover slip and incubated at 37°C and 5% CO_2_ for 1, 2, 3, 4, 6, 8, 12, and 24 h. At these time points, the wells were washed 3x with PBS and 1 ml of methanol was added to each well for 5 min, after which the methanol was removed and plates were dried. Then, the cells were stained with 1 ml of 5% crystal violet for 5 min and then washed 3x with PBS. The dried cover slips were removed from the wells and mounted on a slide with cytoseal 60 (Thermo). Biofilms were imaged at 60x with an Olympus BX53FG Microscope.

### *In vivo* bacterial infection of mice

Female CD-1 ICR mice, age six to eight weeks old, were obtained from Charles River Laboratories. Animals were housed in a specific pathogen-free barrier under biosafety level 2 conditions and allowed to acclimate for 5 days prior to treatment. Forty-eight hours before infection, mice were given water supplemented with 5 g/L of streptomycin and 7% fructose. Food was restricted 12 h before infection and 2 h before challenge, regular sterile water was re-introduced and mice were injected intraperitoneally with cimetidine (50 mg/kg, Sigma). The infection doses were prepared by growing the strains in LB medium overnight at 37°C. The next day, cultures were diluted and grown to an OD_600_ of 1.0. A bacterial suspension was centrifuged and 1 x10^9^ CFUs were re-suspended in 400 μl of PBS and each animal received an equal dose via oral gavage.

During the infection process, the number of bacteria was monitored daily in the fecal pellets. Feces were re-suspended in PBS and bacteria plated for enumeration. Colonization was determined at days 4 and 7 (5 mice per group). For quantification of bacteria in tissues, mice were humanely euthanized following protocols approved by the UTMB institutional animal care and use committee (IACUC). The cecum and colon were void of feces and collected in 15 ml tubes containing PBS and homogenized using the Covidien Precision Disposable Tissue Grinder Systems. The re-suspended feces and tissue homogenates were then serially diluted and plated on MacConkey agar (Difco MaConkey Agar, BD 212123), containing streptomycin (100 μg ml^-^1)(Sigma S9137). After overnight incubation at 37°C, colonies were counted and expressed as either CFU per gram of feces or CFU per organ. Kruskal-Wallis one way ANOVA followed by Dunn’s multiple comparisons test was used for comparisons in the animal experiments to the variance of the populations.

### *In vivo* bacterial competition

The mouse strains used, pretreatment, and infection are described above. The infection dose consisted of 1 x 10^9^ CFU of both the wild-type O104:H4 strain C3493 and O104:H4 Δ*lpfA1* in 400 μl of PBS. During the infection, feces were collected every day as described above. Colonization was determined at days 1, 2, 3 and 4 (4 mice per group). For quantification of bacteria in tissue, mice were humanely euthanized following protocols approved by UTMB IACUC. The cecum and colon were void of feces and collected in 15 ml tubes containing PBS and homogenized using the Covidien Precision Disposable Tissue Grinder System. The re-suspended feces and tissue homogenates were then serially diluted and plated on MacConkey agar containing streptomycin (100 μg ml^-1^). After overnight incubation at 37°C, colonies were counted and expressed as either CFU per gram of feces or CFU per organ. Kruskal-Wallis one way ANOVA followed by Dunn’s multiple comparisons test was used for comparisons in the animal experiments to the variance of the populations.

## Results

### Conservation and classification of *lpfA1* and *lpfA2A* genes among O104:H4 and other pathogenic *E*. *coli* isolates

**Fig 1:**
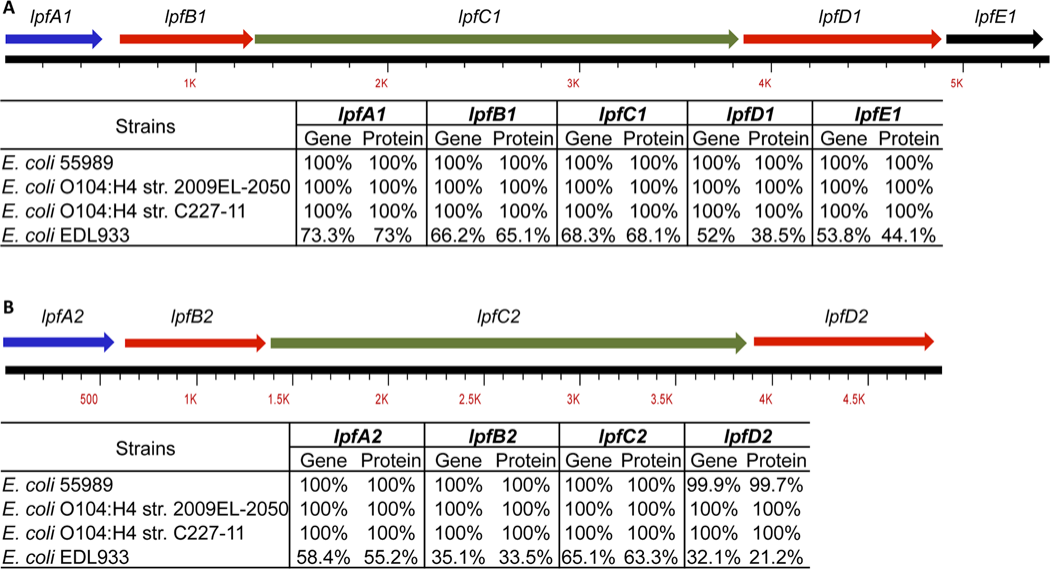
Conservation of *lpf1* and *lpf2* operons within *E*. *coli* O104:H4 and O157:H7. All genes and subsequent proteins from *E*. *coli* O104:H4 2011c-3493 where aligned with EAEC strain 55989, *E*. *coli* O104:H4 2009EL-2050 and O104:H4 C227-11. An alignment was also done with EHEC O157:H7 EDL933.

We examined gene and protein conservation of both O104:H4 2011c-3493 *lpf1* (*A*, *B*, *C*, *D* and *E*) and *lpf2* (*A*, *B*, *C and D*) loci in the 2009 O104:H4 and the another 2011 isolate, C227. We found that all genes and proteins in the various O104:H4 examined were 100% homologous (Fig 1). We also included EAEC strain 55989, isolated from a HIV positive patient with diarrhea in the Central African Republic, because this strain is similar to the 2011 isolates. The operons from EAEC 55989 displayed 100% identity and similarity to the O104:H4 *lpf1* and *lpf2* loci (Fig 1). In contrast, the EHEC EDL933 differed greatly at the gene level (32.1–73.3% homology) and at the protein level (21.2–73% homology) for both *lpf1* and *lpf2* loci (Fig 1). Using the *lpf* gene variant classification proposed by Torres *et al*. [35] to distinguish *lpfA1* and *lpfA2* major fimbrial subunits, we found that O104:H4 *lpf1A* is homologous to the *lpfA1* found in EPEC H30 (O26:H11) and is classified as *lpfA1-2*. In the case of O104:H4 *lpfA2*, it can be classified as *lpfA2-1* due to its homology with O113:H21 *lpfA2* (data not shown).

To assess the distribution of both O104:H4 *lpfA1* and *lpfA2* genes within the genomes of a wide collection of pathogenic *E*. *coli* strains, we performed megablast analysis using 1,167 genomes publically available in the NCBI repository. Interestingly, we found that *lpfA1* was highly conserved within *E*. *coli* O104:H4 strains (Table 2). The *lpfA1* gene was also found in 19.8% of STEC strains, while homologous genes were found in 10% or less of all other groups evaluated. We found that the O104:H4 *lpfA2* gene was conserved within *E*. *coli* O104:H4 strains more frequent than the *lpfA1* gene (Table 2). Homologous O104:H4 *lpfA2* genes were present in 47.6% of avian pathogenic *E*. *coli* (APEC) and 43.8% of antibiotic resistant strains. The remaining groups had 21% or lower percentage of homology with the O104:H4 *lpfA2* gene.

**Table 2:** Distribution of *E*. *coli* O104:H4 *lpfA* gene homologues among different *E*. *coli* strains.

	Total sequences	*lpfA1*	*lpfA2*	% *lpfA1*	% *lpfA2*
**STEC strains**	585	116	126	19.8	21.5
**Antibiotic resistant strains**	48	4	21	8.3	43.8
**APEC and chicken retail meat strains**	21	1	10	4.8	47.6
**UPEC strains**	230	24	38	10.4	16.5
***E*. *coli* O104:H4 strains**	53	50	50	94.3	94.3
**ETEC strains**	230	13	50	5.7	21.7

Note: Both O104:H4 2011c-3493 *lpfA1* and *lpfA2* genes where megablasted against various populations of *E*. *coli* serogroups. The presence of the *lpfA1* and *lpfA2* genes are displayed as percentages of the total sequences examined.

### Contribution of Lpf in adhesion of non-polarized epithelial cells

**Fig 2:**
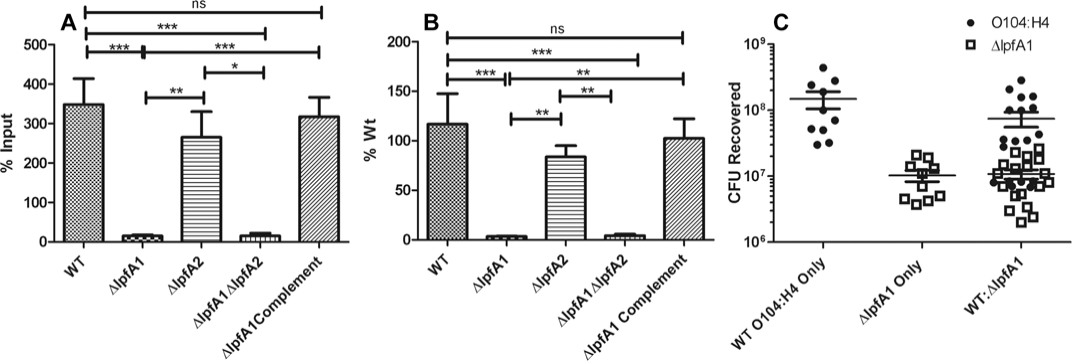
Effect of *lpf1* and *lpf2* mutants on adhesion of *E*. *coli* O104:H4 to differentiated intestinal epithelium. *E*. *coli* O104:H4, Δ*lpfA1*, Δ*lpfA2*, Δl*pfA1* Δ*lpfA2*, and Δl*pfA1* complemented strains’ adhesion was measured in non-polarized Caco-2 cells. All experiments where done with an MOI of 100 and bacteria was recovered and quantified at 3 h post-infection. Results are expressed as (A) percent input and (B) input compared to the wild type strain. Competition of wild type and Δ*lpfA1* was done in the same way and seen in (C) as input of CFUs recovered. Error bars indicated SEM of 3 independent experiments each performed with triplicate wells.

We then evaluated the contribution of Lpf in epithelial cell adhesion. After construction of three isogenic mutants: Δ*lpfA1*, Δ*lpfA2*, Δ*lpfA1* Δ*lpfA2*, and complementation of the Δ*lpfA1* by re-insertion of the *lpfA1* gene, we determined their ability to adhere to Caco-2 cells. Wild-type O104:H4 c3494 strain and the Δ*lpfA2* adhered to the Caco-2 monolayer to numbers higher than the input, (MOI 100), yielding 348.1 ± 198.1% and 265.4 ± 159.0% of the input respectively (Fig 2 panels A and B). Both the Δ*lpfA1* and Δ*lpfA1* Δ*lpfA2* were significantly reduced in their ability to adhere, both binding to only 15.3 ± 9.5% and 15.3 ± 17.2% of their input and 3.4 ± 1.2% and 4.3 ± 3.7% compared to the wild-type strain, respectively (Fig 2 panels A and B). To confirm that the loss of LpfA1 resulted in reduced adherence, we complemented the *lpfA1* mutation and the resulting strain was able to recover the capacity to adhere to a degree comparable to the wild-type strain, adhering to 317.3 ± 120.5% of the input and 102.4 ± 48.7% of wild type (Fig 2 panels A and B).

Further, differences in bacterial growth were evaluated in all the strains and no differences were found, which indicated that the bacteria recovered will not be biased by differences of growth rates after 3 h (S1D Fig). We also evaluated the capacity of the wild-type and Δ*lpfA1* strains to express fimbriae on the bacterial surface. Immunogold EM failed to show specific Lpf1 labeling, because the antisera used was derived from *Salmonella* LpfA and these two fimbria are not highly homologous. However, we were able to demonstrate that fimbriae are still produced on the surface of the wild-type and Δ*lpfA1* strains (S1E and S1F Fig), and since all the strains carry the pAA plasmid, we assume that the fimbriae could correspond to AAF/I.

Finally, to test whether *ΔlpfA1* binding remains reduced in the presence of wild-type strain; we performed an *in vitro* competition assay. We compared the *ΔlpfA1* and wild-type co-culture infections with single strain infections (all wells received at MOI of 100). There was no significant difference in adherence of *ΔlpfA1* or wild-type strains in the competing group compared to the single strain infections (Fig 2 panel C).

### Effect of Δ*lpfA1* and Δ*lpfA2* on adhesion of polarized Caco-2 cells

**Fig 3:**
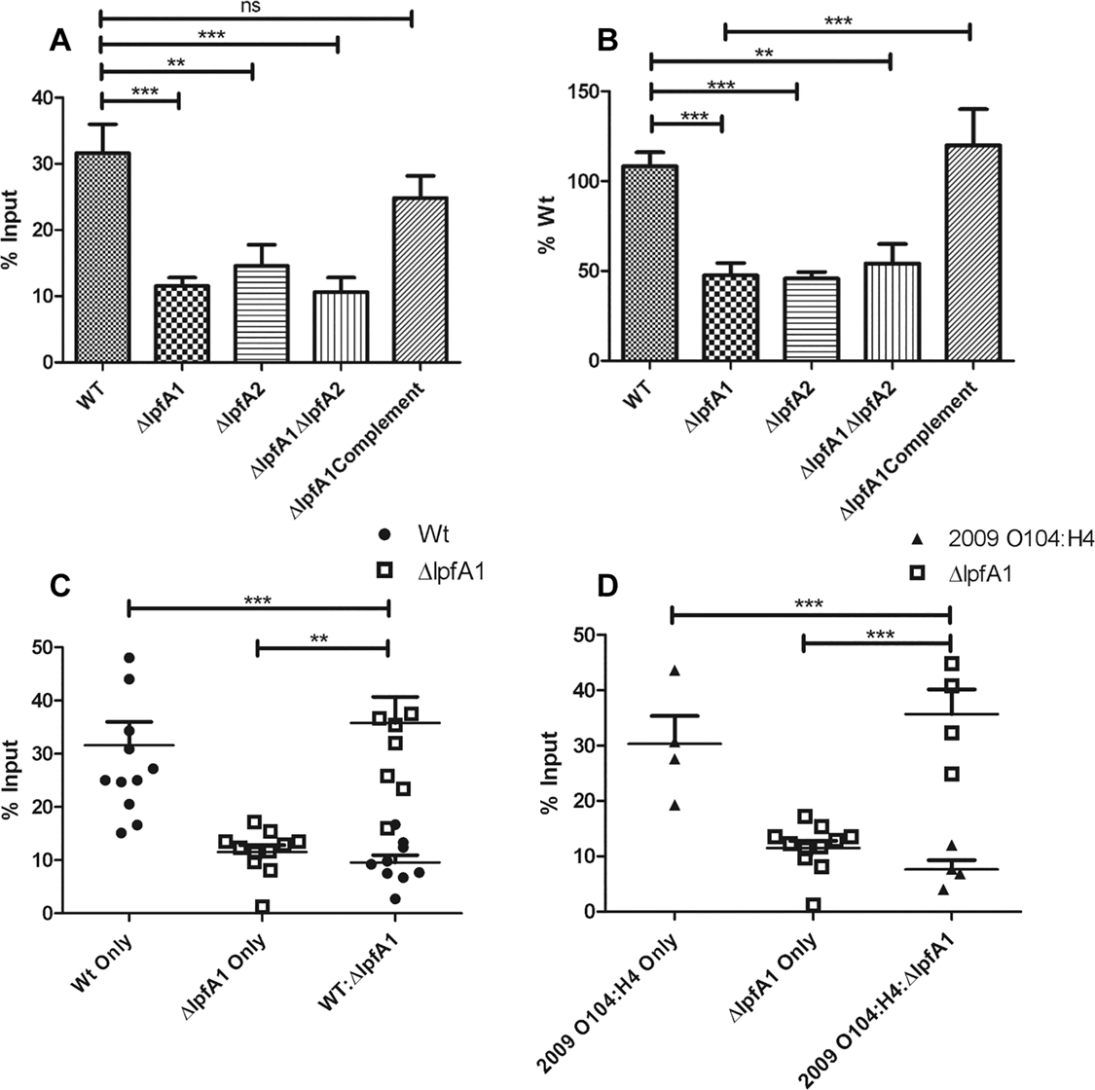
The Effect of *lpfA1* and *lpfA2* mutation on adhesion to polarized epitheial cell. CFUs recovered after a 3 h incubation of wild type O104:H4 2011 c3494, Δ*lpfA1*, Δ*lpfA2*, Δ*lpfA1* Δ*lpfA2*, and Δ*lpfA1* complemented strains were calculated as percent input (A) and input compared to wild type (B). Competition assays of (C) O104:H4 2011 c3494 wild type and Δ*lpfA1* and (D) O104:H4 2009 2050 wild type and O104:H4 c3494 Δ*lpfA1* are expressed as percent input of CFUs recovered. All experiments were done with an MOI of 100 and bacteria was recovered and quantified at 3 h post-infection. Error bars indicated SEM of 2–3 independent experiments each performed with duplicate or triplicate wells.

Next, polarized Caco-2 cells were used to represent a more complex system and more akin to the *in vivo* environment. With the polarized cells, we found that the wild type adhered to 31.6 ± 15.2% of the input (Fig 3 panel A). The *ΔlpfA1*, *ΔlpfA2*, and *ΔlpfA1 ΔlpfA2* mutants had significantly reduced adherence compared to the wild-type strain, adhering to levels 11.5 ± 4.2%, 14.6 ± 8.4%, and 10.7 ± 5.7% of their input and 47.6 ± 22.8%, 45.8 ± 9.8, and 54.1 ± 28.9% of the wild type, respectively (Fig 3 panels A and B). Upon complementation of *ΔlpfA1*, adherence was restored to 120.0 ± 45.0% of the wild type (Fig 3 panel B).

We then tested *Δlpf1*A fitness using a competition assay with the wild-type strain. We measured adherence of wild type and *ΔlpfA1* alone or as a co-cultured. We recovered significantly more *ΔlpfA1* in the competition group compared to wild type (Fig 3 panel C). We recovered 31.6 ± 15.2% of the wild-type strain, 10.2 ± 4.1% of *ΔlpfA1* in the single infections and 9.6 ± 4.1% and 33.1 ± 17.1% of the wild-type and *ΔlpfA1* strains in the co-cultured infections, respectively (Fig 3 panel C).

Finally, the competition capacity of the O104:H4 c3494 *ΔlpfA1* was also evaluated against *E*. *coli* O104:H4 strain 2050, which is an EAEC/STEC strain isolated from the Republic of Georgia in 2009. As noted above, these two *E*. *coli* O104:H4 strains have identical *lpf1* loci. Similar adherence results were found as previously described, with recovery of 35.7% of the input of *ΔlpfA1* and only 7.6% of the O104:H4 2050 wild type from co-cultured cells (Fig 3 panel D). In the wells infected with the individual strains, 20.2 ± 10.1% of O104:H4 2050 was recovered and 11.2 ± 3.3% of the O104:H4 *ΔlpfA1* input (Fig 3 panel D). In both competition environments, the *ΔlpfA1* strain was able to out-compete the wild type strains.

### *lpfA1*’s contribution to formation of stable biofilm

**Fig 4:**
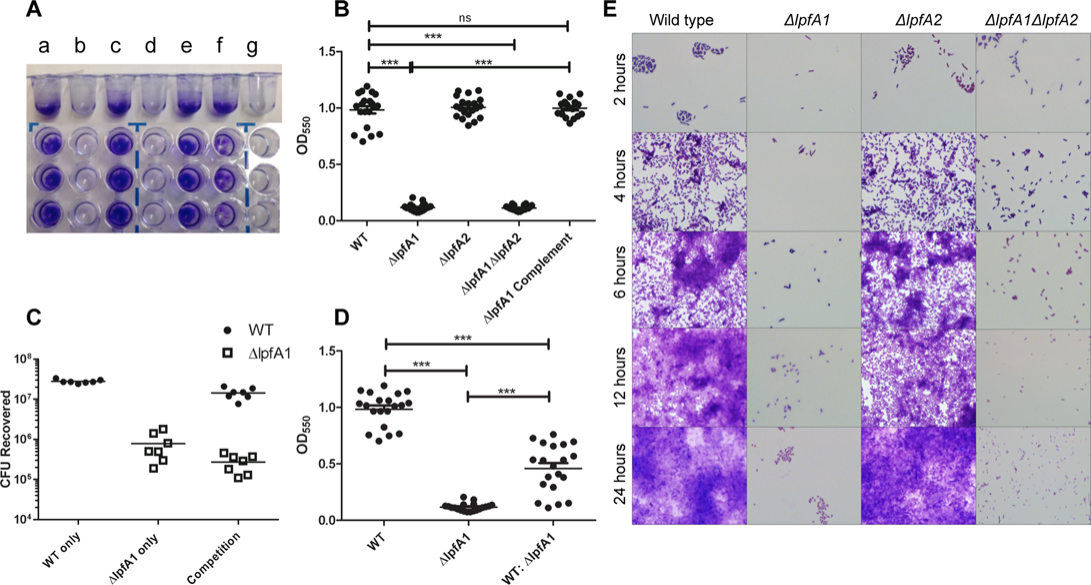
Δ*lpfA1* and Δ*lpfA2*’s effect on stable biofilm formation. Microtiter plates were washed and stained with crystal violet and biofilms visualized in (A) and quantified in (B) of *E*. *coli* O104:H4 (a), Δ*lpfA1* (b), Δ*lpfA2* (c), Δ*lpfA1* Δ*lpfA2* (d), Δ*lpfA1* complemented (e), O104:H4 wt and Δ*lpfA1* mix (f) or MEM media alone (g) after 24 h of static incubation at 37°C. Competition assays were done with O104:H4 2011 c3494 wild type and Δ*lpfA1* mutant, and results are expressed as absorbance at 550 nm (C) and CFUs recovered (D). Formation of biofilm by all *E*. *coli* O104:H4 wild type and mutant strains were visualized by growing on glass cover slips, stained and imaged at 2, 4, 6, 12, and 24 h post-infection (E). All experiments were done with a 1:100 dilution of overnight cultures diluted to and OD_600_ of 1.0. Error bars indicated SEM of 3 independent experiments each performed with triplicate wells.

Since biofilm formation is a hallmark characteristic of EAEC strain’s colonization, we tested whether Lpf1 and Lpf2 were required for stable biofilm formation. Wild type O104:H4 forms the most stable biofilm in DMEM and poorly in LB, MacConkey broth, or EMEM (data not shown), so biofilm formation was tested in DMEM. Upon static growth in polystyrene plates, we found that *ΔlpfA2* mutation did not cause reduction in biofilm formation and this was represented by the absorbance of 1.00 ± 0.09 compared to 0.98 ± 0.86 for the wild type strain (Fig 4 panels A and B). Both the *ΔlpfA1* and *ΔlpfA1 ΔlpfA2* strains were unable to form a stable biofilm, yielding optical readings of 0.12 ± 0.03 and 0.11 ± 0.02, respectively (Fig 4 panels A and B). Upon complementation of the *ΔlpfA1* gene, biofilm formation was restored and optical readings went back to 0.99 ± 0.08. A competition assay was performed with the wild type strain and Δ*lpfA1* to determine whether the mutant strain could be incorporated into the biofilm and at which extent the absorbance and CFU enumeration was modified. We compared biofilms created by individual wild-type O104:H4 and *ΔlpfA1* strains and those resulting from equal amounts of the combined strains. The competition group resulted in an average absorbance reading of 0.46 ± 0.21, which was significantly lower than the wild type (0.98 ± 0.86); however, the number of wild-type CFU incorporated into the biofilm was not significantly reduced (2.4 x 10^7^ ± 2.3 x 10^7^) as compared to the wild-type biofilm (2.8 x 10^7^ ± 2.9 x10^6^) (Fig 4 panels C and D). For the *ΔlpfA1* individual biofilm, there was no significant change in the bacteria recovered since we cultured 2.7 x 10^5^ ± 1.3 x 10^5^ Δ*lpfA1* bacteria from the competition group and 7.8 x 10^5^ ± 6.0 x 10^5^ from the *ΔlpfA1* only biofilm (Fig 4 panel D).

To determine if the *ΔlpfA* mutant strains where unable to bind to the surface or unable to form the brick stacking pattern characteristic of EAEC strains, we visually monitored biofilm formation over a 24 h period. We collected coverslips with bacterial growth at 1, 2, 3, 4, 6, 12, 18, and 24 h post-infection (Fig 4 panel E). Once again, the *ΔlpfA2* mutant displayed a pattern similar to the wild-type strain, forming organized aggregates by 2 h and a more complex pattern by 3 h. After this time point, wild type and *ΔlpfA2* began stacking to form a multilayer biofilm and by 4 h, they had substantial production of stacking (Fig 4 panel E). The *ΔlpfA1* and *ΔlpfA1 ΔlpfA2* only had small numbers of bacteria bound to the plastic surface and virtually had no variation in binding over time. One thing to note was that both strains formed a non-homogenous suspension with weak aggregations in the liquid suspension, which differed from the fully homogenized suspension of the wild-type strain and the *ΔlpfA2* strains (data not shown).

### Altered *In vivo* colonization

**Fig 5:**
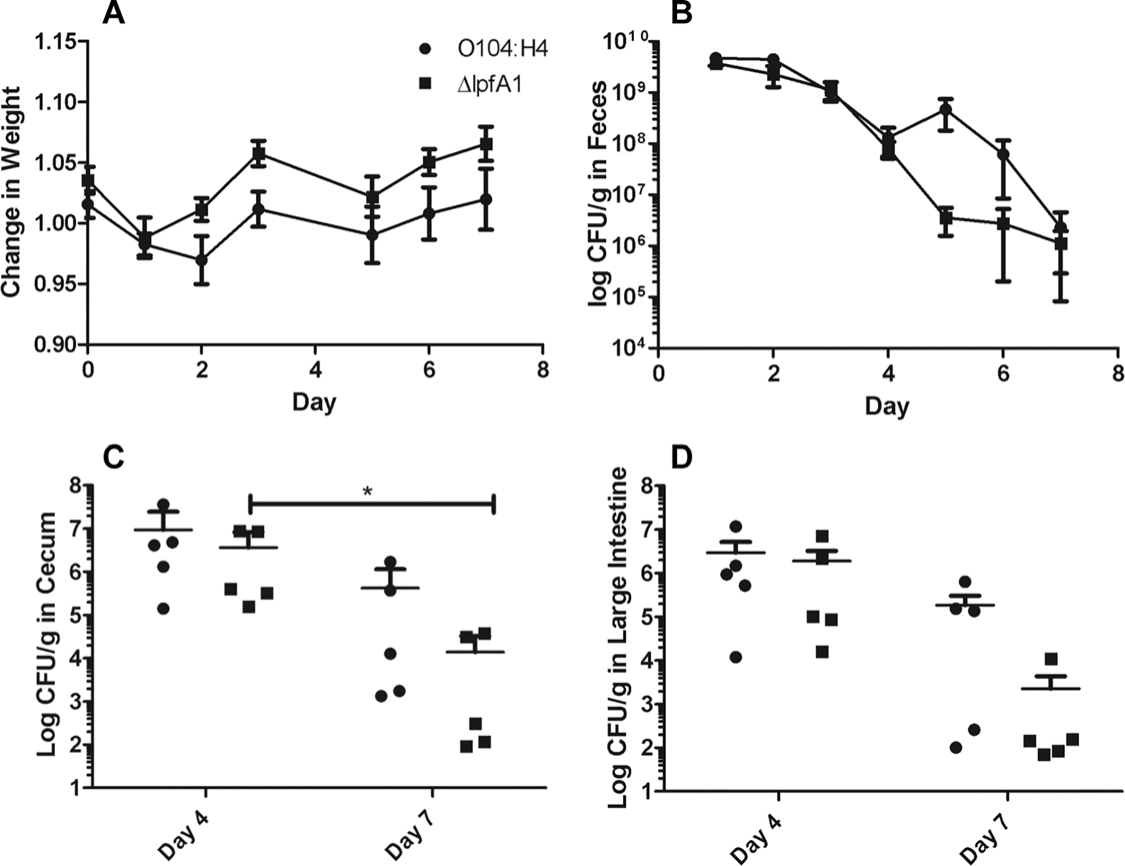
*In vivo* colonization of mice by wild type O104:H4 and *ΔlpfA1*. Six to eight week old CD-1 ICR mice were infected with 1x10^9^ O104:H4 2011 c3494 wild type (n = 10) or Δ*lpfA1* (n = 10). Their weight (A) fecal shedding (B; expressed as log CFUs/g) were monitored for 7 days. On days 4 and 7, ceca (C) and large intestines (D) where collected and bacteria recovery is expressed as log CFU/g of tissue. Error bars indicated SEM of 5 or 10 mice.

CD-1 ICR mice were inoculated via gavage with 1 x 10^9^ CFU of O104:H4 c3493 or *Δlpf1A* strains. The wild-type strain resulted in a slight but not significant change in weight starting 2 days post infection while compared to *ΔlpfA1* strain (Fig 5 panel A). The fecal shedding of the wild type strain was increased between days 4 and 7 with a peak at 5 days post infection (Fig 5 panel B). Upon organ collection at day 4, we found no significant difference in the numbers of wild-type and *ΔlpfA1* bacteria collected from the cecum and large intestine (Fig 5 panels C and D). On day 7 post infection, ceca and large intestines were collected and although we did not observe a significant difference in the numbers of the wild-type and *ΔlpfA1* strains recovered, we found a significant decrease in Δ*lpfA1* collected from the ceca between days 4 and 7, suggesting faster clearance of the *ΔlpfA1* strain from the cecum.

### *In vivo* competition of wild type O104:H4 and *ΔlpfA1*

**Fig 6:**
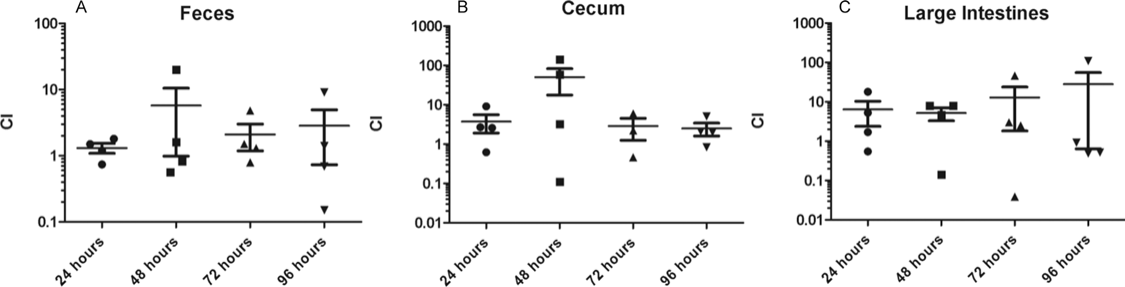
Competitive index *in vivo* of wild type O104:H4 and Δ*lpfA1* strains. Six to eight week old CD-1 ICR mice where infected with 1x10^9^ O104:H4 2011 c3494 wild type (n = 12) and Δ*lpfA1* (n = 12) totaling 2x10^9^ CFU. Collections were done at 24 (solid circles), 48 (solid squares), 72 (solid triangles), and 96 h (inverted triangles). Fecal shedding (A), ceca (B), and large intestine (C) recoveries of both strains are expressed as Competitive Index (CI = % Δ*lpfA1* recovered / % wild type recovered). Error bars indicated SEM of 4 mice.

CD-1 ICR mice were inoculated with equal amounts of wild type O104:H4 and Δ*lpfA1*, totaling 2 x 10^9^ CFU. Every day for 4 consecutive days, feces, cecum, and large intestine were collected from 4 animals, and results were expressed as competitive index (Δ*lpfA1* / wild type). We found that wild type did not out-compete the Δ*lpfA1* strain (Fig 6 panels A-C). Instead, we recovered significantly higher numbers of Δ*lpfA1* compared to wild type, similar to what we observed in the *in vitro* competition assay with polarized cells. At all-time points tested, feces, ceca, and large intestine collections yielded higher numbers of Δ*lpfA1* strain, with the largest differences at 48 h in the cecum and at all-time points in the large intestine (Fig 6 panels A-C).

## Discussion

It is important to understand how *E*. *coli* O104:H4 colonizes the intestine with the goal of combating and eventually preventing bacterial intestinal binding as an alternative treatment option. We decided to focus on the study of the initial stages of adhesion, which might impact further colonization events. In the presented work, we have investigated the role of the Lpf1 and Lpf2 in colonization of the German *E*. *coli* O104:H4 2001c-3494 isolate. Belonging to the EAEC classification, this strain produces a biofilm, which represents a complicated microenvironment with the possible involvement of multiple adhesins. In order to test the role of Lpf1 and Lpf2, we generated isogenic single *lpfA1*, *lpfA2*, and double *lpfA1 lpfA2* mutant strains. To elucidate whether these fimbriae play a role in bacteria-bacteria or bacterial-host interactions, we tested their ability to adhere to intestinal cells both *in vitro* and *in vivo*, as well as determined if they are necessary for the formation of a stable biofilm.

Starting with the role of Lpf2, we found that loss of functional *lpf2* did not affect the strain’s ability to form a confluent biofilm, nor did the gene loss affect its ability to colonize non-polarized Caco-2 cells. The Δ*lpf2* did, however, have significant reduction in its ability to adhere to polarized Caco-2 cells. This suggests some role in adherence, perhaps dependent on the expression of specific host receptors on the apical side of the cells. Due to the fact that Δ*lpfA2* only had partially lost function, we did not further study the strain in an animal model; instead we focused on the Δ*lpf1* strain which had a much greater degree of impairment.

The loss of functional Lpf1 resulted in substantial reduction in binding capabilities of the O104:H4 strain, similar to what we previously reported for EHEC O157:H7 Lpf1 [32]. *E*. *coli* O104:H4 *ΔlpfA1* had reduced ability to adhere to either *in vitro* model, as well as to form a biofilm. The *ΔlpfA1* was also examined with electron microscopy, which showed the presence of surface fimbrial appendages, supporting the potential role of other fimbriae during colonization of the intestine. The *ΔlpfA1* mutant did not have significant reduction in the *in vivo* colonization model, but showed a trend of lower bacterial recovery from the feces, ceca, and large intestine. This finding reiterates that O104:H4 has multiple factors that aid in adhesion, a similar phenomenon, which was observed when we mutagenized both *lpf* operons in EHEC O157:H7 [27,28]. However, our results with the *lpfA1* mutant supported the idea that Lpf1 is critical for effective colonization of the intestine by *E*. *coli* O104:H4, even in presence of the AAF/I fimbriae.

In addition to exploring the traditional role of fimbriae in bacterial adhesion and knowing that some adhesins (particularly Lpf1) are associated with tissue specificity [22,29], we decided to further examine whether Δ*lpfA1* mutant colonization was restored while in presence of the wild-type strain. This was done to help elucidate whether Lpf1 works as a bacteria-bacteria or as bacteria-host adhesin. Interestingly, the *lpf1* mutant out-competed the wild-type strain in the polarized Caco-2 cell model, as well as in the *in vivo* murine model of infection. This suggests that *lpfA1* mutant expressed other adhesins that mediate bacteria-bacteria interactions. Alternatively, it is plausible to propose that the wild-type strain is used as platform for the Δ*lpfA1* mutant to bind, which increases its interactions with the putative host receptor in the epithelia.

Taking this data and applying it to other Lpf-positive strains based on conservation of *lpf1* and *lpf2* loci, we can speculate that the function of Lpf1 in adhesion is not only limited to the strains of *E*. *coli* O104:H4 isolated in 2011, but to the 2009 strain 2050 and the EAEC strain 55989. Interestingly, we expanded our analysis and found that the *lpfA2* is more widely distributed than *lpfA1* in a wide variety of pathogenic *E*. *coli* strains. Although a significant number of further experimentation is needed, it is plausible to speculate that therapeutic treatment targeting the function of Lpf1 rather than Lpf2 could be more effective in reducing colonization, as well as re-colonization by bacteria dispersed from a mature biofilm [13,14].

We also confirmed that the experimental results obtained are dependent on the *in vitro* adhesion model used because it was found that Δ*lpfA1* could not out-compete wild-type strain when using non-polarized Caco-2 cells. This is in agreement with the fact that the polarized cells mimic more closely the *in vivo* environment due to their ability to form apical and basolateral sides [36,37]. To date, the presence and/or difference in Lpf receptor localization has not been elucidated, but the findings in our study suggested that wild-type strain might be able to contact the putative receptors expressed in these models (polarized cell assay and *in vivo* murine infection). The presence of alternative bacterial adhesins or host receptors might facilitate the Δ*lpfA1* mutant to bind and get trapped between the wild-type bacteria and the host mucosa or within the co-cultured biofilms. In the case of EHEC O157:H7, we have previously demonstrated that increased curli expression occurred upon mutation of both *lpf1* and *lpf2* operons [27,28], and several pathogenic *E*. *coli* strains compensate for the absence of an adhesin with expression of other colonization factors [22,38]. Based on our *in vitro* and *in vivo* results, we can conclude that the Lpf1 adhesin expressed by *E*. *coli* O104:H4 participates in bacteria-host cell adherence, and that the Lpf1 fimbriae are responsible for initial events of intestinal binding, even in presence of the AAF/I fimbriae.

## Supporting Information

S1 Fig Isogenic mutants confirmation and growth curve. PCR confirmation for *lpfA1* (A), *lpfA2* (B), and of *aggA* (C) was done on all strains (+ = wt, Δ1 = Δ*lpfA1*, Δ2 = Δ*lpfA2*, Δ1Δ2 = Δ*lpfA1* Δ*lpfA2*, and Δ1C = Δ*lpfA1* complemented). Growth kinetics were done on all strains in comparison to the wild type O104:H4 2011 c3494 to check for altered growth rates of the mutants. The growth curve was done twice and (D) is a representative of the two independent experiments. Immunogold-labeling electron microscopy of wild type (E) and Δ*lpfA1* (F) strains. Arrows depict surface fimbrial appendages.
(TIF)
